# Development of Magnetic Torque Stimulation (MTS) Utilizing Rotating Uniform Magnetic Field for Mechanical Activation of Cardiac Cells

**DOI:** 10.3390/nano10091684

**Published:** 2020-08-27

**Authors:** Myeongjin Song, Jongseong Kim, Hyundo Shin, Yekwang Kim, Hwanseok Jang, Yongdoo Park, Seung-Jong Kim

**Affiliations:** 1 Department of Biomedical Sciences, College of Medicine, Korea University, Seoul 02841, Korea; myungjinsong@korea.ac.kr (M.S.); envokim72@korea.ac.kr (J.K.); rladprhkd12@korea.ac.kr (Y.K.); kevin14@korea.ac.kr (H.J.); 2 Department of Mechanical Engineering, Yonsei University, Seoul 03722, Korea; shinhd83@nate.com

**Keywords:** magnetogenetics, mechanotransduction, torsional magnetic stimulation, cardiac cells, hypoxia

## Abstract

Regulation of cell signaling through physical stimulation is an emerging topic in biomedicine. Background: While recent advances in biophysical technologies show capabilities for spatiotemporal stimulation, interfacing those tools with biological systems for intact signal transfer and noncontact stimulation remains challenging. Here, we describe the use of a magnetic torque stimulation (MTS) system combined with engineered magnetic particles to apply forces on the surface of individual cells. MTS utilizes an externally rotating magnetic field to induce a spin on magnetic particles and generate torsional force to stimulate mechanotransduction pathways in two types of human heart cells—cardiomyocytes and cardiac fibroblasts. Methods: The MTS system operates in a noncontact mode with two magnets separated (60 mm) from each other and generates a torque of up to 15 pN µm across the entire area of a 35-mm cell culture dish. The MTS system can mechanically stimulate both types of human heart cells, inducing maturation and hypertrophy. Results: Our findings show that application of the MTS system under hypoxic conditions induces not only nuclear localization of mechanoresponsive YAP proteins in human heart cells but also overexpression of hypertrophy markers, including β-myosin heavy chain (βMHC), cardiotrophin-1 (CT-1), microRNA-21 (miR-21), and transforming growth factor beta-1 (TGFβ-1). Conclusions: These results have important implications for the applicability of the MTS system to diverse in vitro studies that require remote and noninvasive mechanical regulation.

## 1. Introduction

Mechanical force and its use in mediating molecular functions, cell signaling, and behavioral responses are pivotal in the regulation of complex biological systems [1,2,3,4]. During biological processes, mechanoreceptors adopt and utilize this force to accomplish their mechanisms, suggesting the possibility of using mechanical force to regulate biological pathways [5,6]. While the rapid progress in the development of force stimulation systems has improved the understanding of mechanotransduction processes [7,8,9], the use of these systems in various applications is currently challenging due to their short working distances and/or contact-requiring modes of action [10,11]. However, application of these systems is necessary not only to facilitate laboratory investigations of in vitro systems but also to develop translational research for health technology innovations [10,11,12]. To this end, an innovative framework that incorporates a widely adaptable and reliable force-stimulating system for modulating cellular functions is highly required, as shown in optogenetics systems that combine an optical stimulator with a light-responsive ion channel (i.e., channelrhodopsin) [13].

During the past decade, magnetogenetics has emerged as a method to provide noninvasive mechanical stimulation, because magnetic fields freely penetrate all parts of the body [14,15,16,17], However, current methods of magnetic stimulation by a mechanical pulling force have an insufficient working range for diverse biomedical applications that require a distance of more than a few centimeters [18]. For instance, the penetration depth for cardiac stimulation should be at least ~60 mm to target the human heart by magnetic stimulation [19]. In addition, simultaneous equivalent modulation of thousands of cardiac cells or organoids by a magnetic force would facilitate investigation of their maturation and function in 2D and 3D [20,21]. This observation implies that the capability for long-distance and multiple stimulations would augment the intrinsic merits of magnetic systems as candidates for noninvasive mechanical stimulation systems [22].

Development of novel magnetic nanomaterials is a prerequisite for advancing magnetic modulation systems in biological applications [23]. To utilize the merit of magnetic modality, magnetic nanomaterials have been developed by enhancing their properties including magnetism, size, and tunability [24]. In addition, hybrid nanomaterials composing iron oxide and other compounds have been investigated, which allows applying a magnetic system for modulation of cell behavior, drug delivery, and bioimaging [25,26,27]. Such hybrid magnetic nanomaterials can provide a unique and additional feature to the canonical magnetism, which is exclusively important for biological applications.

Cardiomyocytes and cardiac fibroblasts are examples of mechanically coupled cells that obtain cues from the mechanical environment for their physiological development and homeostasis [28,29,30]. To date, numerous methods for mechanically stimulating cardiomyocytes and cardiac fibroblasts have been demonstrated to identify the importance of mechanical stimulation in cardiac systems [31]. The methods are classified by their use of passive or active modalities to transduce mechanical forces into cardiac cells [30]. In passive systems, cardiac cells entrapped in matrix gels are hung between rods, and the intrinsic force originating from their beating behavior results in their maturation and arrangement [32]. Alternatively, cardiac cells are placed on cyclically stretched substrates that have also been shown to enhance cardiac maturation [33,34]. Magnetic nanoparticles and microparticles have been utilized to apply attractive magnetic forces to biological systems such as cells and tissues [35]. However, the use of such attractive forces is insufficient for long-distance applications due to limitations of their action mode.

**Figure 1:**
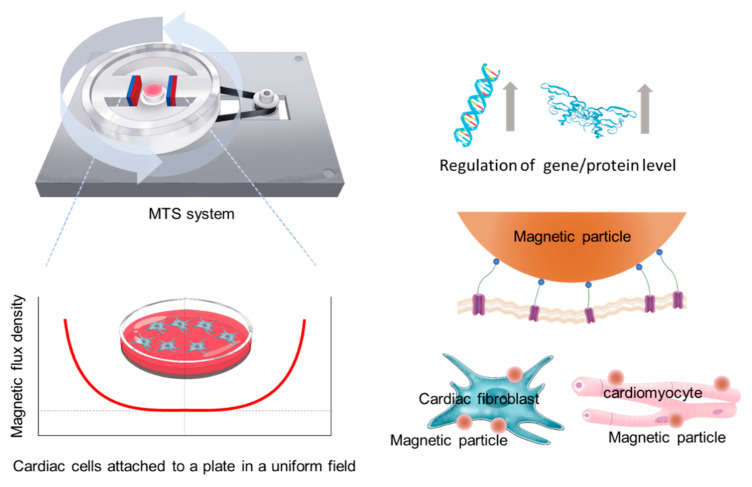
Schematics of magnetic torque stimulation (MTS) system (**left**) and its application for mechanostimulation of cardiac cells (**right**).

Here, we developed a magnetic torque-stimulation (MTS) system that generates a torsional force to rotate magnetic particles (MPs) at a biological interface, facilitating the stimulation of mechanotransduction pathways in vitro (Figure 1). The MTS system is composed of wheat germ agglutinin (WGA)-conjugated MPs (CMPs) and a rotating magnetic field (RMF), which enables the generation of a highly uniform torsional force by each CMP on the cell surface over a long distance. Since the torque generated by a rotating magnetic field is dependent mainly on the magnetic field strength or magnetic flux density (B), the generation of a sufficiently uniform magnetic flux density (>200 mT) covering a wide area is crucial for remote stimulation. Our MTS system can cover a wide circular area (35 mm in diameter), yielding the ability for remote force loading of up to 30 pN by a single CMP (2.8 µm). By computationally optimizing the rotating magnetic field using COMSOL™ with two-dimensional (2D) finite element analysis (FEA), we built a highly uniform MTS system for remotely stimulating mechanotransduction pathways in AC16 human cardiomyocytes (AC16-hCMs) and human cardiac fibroblasts (hCFs). The stimulation results suggested that the MTS-CMP system is active over long distances to mediate gene expression and protein localization in AC16-hCMs and hCFs to promote their maturation and hypertrophy.

## 2. Materials and Methods

### 2.1. MTS Assembly

The conceptual overview of the developed MTS system and its application for mechanostimulation of cardiac cells are shown in Figure 1. This system aimed to enhance not only the strength but also the uniformity of the magnetic field across a target region. The system has a pair of rectangular permanent magnets (PMs) facing each other and located 6 cm apart that are attached to the inside of a circular back yoke. A cell culture dish lies in the target region between the PMs. The PMs used are made of neodymium with a remanent flux density of 1.3 T and a relative permeability of 1.05. The back yoke, which provides the outer magnetic flux path, is made of low-carbon steel. The PMs and yoke are rotated by a geared motor (GM60-3657-2439, DC 24V, gear ratio: 1/10) with a timing belt that is mounted under the base plate. A motor controller (Q8-42S) controls the rotation speed at ~60 rpm that is selected due to the similarity to the heartbeat. The entire system is designed to withstand the high humidity of an incubator.

### 2.2. Cell Culture

AC16 (a human cardiomyocyte cell line; Merck Millipore, Burlington, MA, USA) cells were cultured in Dulbecco’s modified Eagle’s/F-12 medium (Sigma, St. Louis, MO, USA) containing 2-mM L-glutamine (EMD Millipore, Burlington, MA, USA), 12.5% FBS (EMD Millipore, Burlington, MA, USA) and 1× penicillin–streptomycin solution (EMD Millipore, Burlington, MA, USA). hCF (a human normal cardiac fibroblast-ventricular cell line; Lonza, Basel, Switzerland) cells were cultured in fibroblast basal medium (FBM; Lonza, Basel, Switzerland) containing 1× insulin, 1× hFGF-B, and 1× GA-1000 (1× FGM™-3 SingleQuots™ supplement pack, Lonza, Basel, Switzerland). Cells were maintained in a humidified incubator (Panasonic, Osaka, Japan) at 37 °C and 5% CO_2_. Hypoxic conditions for cells were induced by using 100 µM cobalt chloride (CoCl_2_, Sigma, St. Louis, MO, USA), a chemical inducer of hypoxia-inducible factor-1 (HIF-1).

### 2.3. WGA-Coated MPs and MTS Stimulation

For MTS stimulation experiments, AC16 and hCF cells were seeded at a density of 1.5 × 10^5^ cells/dish in a 35-mm cell culture dish and incubated for 24 h before MPs binding. To attach MPs capable of reacting to the MTS system to the cell, we utilized a commercially available MP (2.8 µm; Dynabeads^®^ M-270 Carboxylic Acid, Invitrogen, Carlsbad, CA, USA) that is composed by superparamagnetic nanoparticles. It is worth noting that the superparamagnetic property exclusively belongs to the nanoparticles, is composed of a single magnetic domain, and thus is normally below 50 nm in diameter. This superparamagnetic nanoparticle assembled MPs enable us to apply magnetic torque only when the uniform magnetic field of the MTS system is rotated around a cell culture dish. The MPs were conjugated with WGA (a lectin from *Triticum vulgaris*, Sigma, St. Louis, MO, USA) using the *N*-(3-dimethylaminopropyl)-*N*′-ethylcarbodiimide hydrochloride (EDC, Sigma, St. Louis, MO, USA) coupling method that chemically crosslinked carboxylic groups of MP to amine groups of WGA. First, MPs (1.5 × 10^6^) were washed with 0.1 mM MES buffer (Biosolution, South Korea, Korea). EDC and N-hydroxysuccinimide (NHS, Sigma, St. Louis, MO, USA) were dissolved in 0.1-M MES buffer at concentrations of 0.2 and 0.4 mg/µL, respectively, and incubated for 5 min at room temperature. Then, the washed MPs were mixed with 2 µL of WGA (25 µM) and 1 µL of EDC-NHS solution and reacted at room temperature for 1 h. After incubation, the MPs were washed with medium five times to remove unreacted substances. Cells that were attached to a 35-mm cell culture dish and incubated for 24 h were seeded with WGA-coated MPs in a humidified incubator (Panasonic, Osaka, Japan) at 37 °C and 5% CO_2_ for 20 min. After incubation, the cells were gently washed with the corresponding medium three times and incubated under each experimental condition, i.e., MPs only (control), MF only, hypoxia, and MF-MPs under hypoxic conditions, for 24 h. Note that the stimulation experiment was performed in a humidified incubator at 37 °C and 5% CO_2_.

### 2.4. Immunofluorescence Assay

For immunostaining, cells were fixed with 4% paraformaldehyde (PFA, Biosesang, Seongnam, Korea) for 20 min at room temperature, permeabilized with 0.4% Triton X-100 (Sigma, St. Louis, MO, USA), and blocked with 5% bovine serum albumin (BSA, Sigma, St. Louis, MO, USA). An antibody specific for yes-associated protein (YAP, Santa Cruz Biotechnology, Dallas, TX, USA) was added to the cells (1:200). After the cells were washed with PBST solution (PBS buffer containing 0.05% Tween 20), Alexa 488-conjugated phalloidin (1:200, Life Technologies, Carlsbad, CA, USA) and DAPI (1:3000, Sigma, St. Louis, MO, USA) were added for staining. Fluorescence images were acquired by using a fluorescence microscope (Nikon, Tokyo, Japan). The YAP protein abundance was quantitatively estimated by the intensity histogram obtained from the fluorescence images using ImageJ 1.51w (Java 1.8.0_66, National Institutes of Health, Bethesda, MD, USA).

### 2.5. RNA Isolation and RT-PCR Analysis

Total RNA was isolated from cells with ReliaPrep™ RNA Miniprep systems (Promega, Madison, WI, USA) according to the manufacturer’s protocol. Then, 1 µg of isolated total RNA was reverse transcribed using a PrimeScript RT reagent kit (Takara, Tokyo, Japan). Amplification and detection of specific products were performed in an ABI 7500 real-time system (Applied Biosystem, Foster City, CA, USA). The expression of the β-actin gene was determined as the internal control. The sequence of each primer is listed in Table S1. qRT-PCRs were conducted in duplicate, and the signal was recorded at the end of each cycle. The expression of each gene was calculated as the fold change by comparing the cycle threshold (Ct) values for the β-actin gene and the mRNAs of interest.

### 2.6. Statistical Analysis

All data were analyzed by using Prism version 8.0.2 (GraphPad, San Diego, CA, USA). Statistical analysis was performed by using Student’s *t*-test. *p*-values of <0.05 were considered statistically significant. 

## 3. Results

To develop an MTS system applicable for highly uniform mechanical stimulation in noncontact mode, we first performed magnetic field simulation via 2D FEA with the commercial software COMSOL™. By varying the distance between two magnets attached to a circular back yoke, we constructed a magnetic field strength map (Figure S1). A magnet-to-magnet distance of 6 cm was suitable for generating a uniform magnetic field with a strength of ~200 mT throughout the whole area of a cell culture dish (35 mm), while distances of 4 and 8 cm were not successful in generating a uniform magnetic field (ΔB *>* 5 T/m) and a magnetic field of a sufficient strength (<100 mT), respectively. In general, the greater is the magnetic field strength, the greater is the magnetic force, which is achievable by using stronger magnets and reducing the distance between two magnets. Since a permanent magnet made of neodymium is the strongest one in the market and too short distance between the magnets impairs the uniformity of magnetic field in the working area, the magnetic field strength of ~200 mT and its corresponding torque were almost the maximums we could obtain. We observed the rotation of magnetic bead strings arranged in a row when exposed to the rotating magnetic field (60 rpm) generated by the MTS system (Figure S2). These results suggest that the MTS system can cover a 35-mm-diameter circular region depending on the magnet configuration, providing the possibility of highly parallel yet uniform mechanical stimulation of many cells simultaneously.

In general magnetic theory, the magnetic force and torque can be calculated based on the Maxwell stress tensor method [36], but the conventional formulations are not suitable for calculating the torque acting on a magnetic bead in a rotating magnetic field. Thus, we adopted the modified formulation with an experimental coefficient to calculate the maximum torque, as follows:

(1) τ=N×B

**Figure 2:**
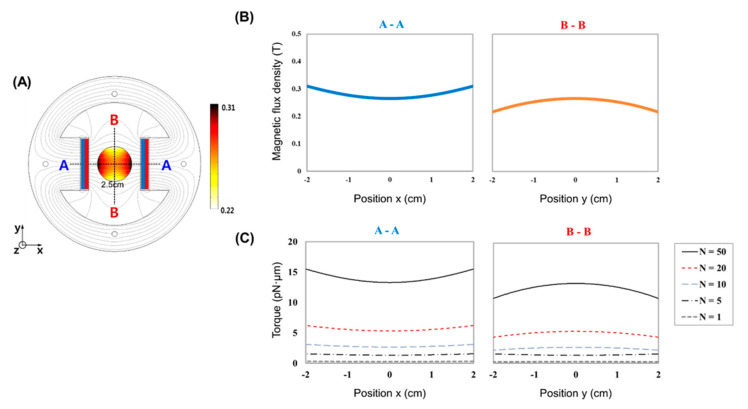
Numerical simulation of MTS system for mechanical stimulation of cells. Configuration of MTS with induced normalized magnetic flux density distribution and the stream line plots (**A**). Cross line plots in x- and y-directions, respectively (**B**). Normalized magnetic flux density field from the simulation result and the torque induced by the magnetic beads using the Equation (1) with the proposed coefficient (**C**).

*N* = *αM_b_* (2)

where *N* is the experimental coefficient and *B* is the magnetic flux density. In the traditional formula, the proposed coefficient *N* can be described by Equation (2) [37,38]. *α* and *M_b_* are the anisotropic magnetic coefficient and magnetization coefficient, respectively. Essentially, the relation represents the interaction between the magnetic field and the magnetic beads. The magnetic torque acting on the beads can be estimated by the proposed coefficient *N* (Figure 2), and *N* can be experimentally determined to meet the characteristic properties of the magnetic beads and the magnetic tweezer system.

Computational simulation by 2D FEA suggested that the MTS system is suitable for generating a quite uniform magnetic field across a target area (Figure 2) that is representative of a 35-mm cell culture dish. The uniform field means that there is no magnetic flux density gradient causing the attractive magnetic force. Importantly, as the anisotropy of the MPs is increased, the torque rotating them toward the direction of the magnetic field increases. When the uniform magnetic field rotates, the MPs are not pulled in either direction but instead rotate in place. If 3-µm magnetic beads are used, each bead can apply ~45 pN of torsional force to the cell surface. The number of MPs attached to each cell was analyzed (Figure S3). This force has been reported to be sufficient to activate numerous mechanotransduction pathways [39].

**Figure 3:**
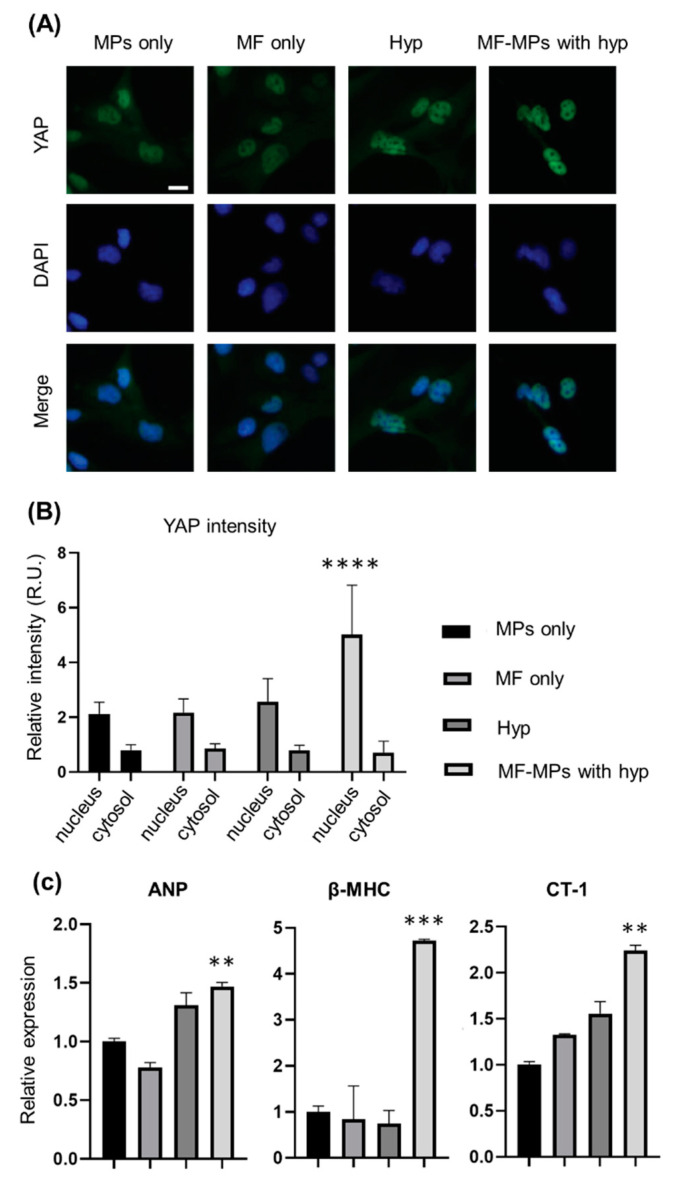
Effects of forces generated by the MTS system on protein localization and gene expression in AC16 cells. Representative fluorescence images of AC16 cells in the groups: MPs only (control), MF only, hypoxia, and hypoxia with MF-MPs for 24 h (scale bars: 100 µm) (**A**). Immunofluorescence images of staining for YAP (green) and the nuclear marker DAPI (blue) showed that YAP translocated from the cytosol to the nucleus. Quantification of YAP expression in the cytosol and nucleus of cultured AC16 cells of each group (**B**). Quantitative mRNA expression data for genes related to cardiac hypertrophy in cultured AC16 cells of each group analyzed by RT-PCR (**C**). A *t*-test was used to compare the control and MF-MPs with Hyp groups (****, *p* < 0.0001; ***, *p* < 0.001; **, *p* < 0.01). There were two independent experiments, and RT-PCR analysis was duplicated.

To investigate whether the MTS system can stimulate a mechanotransduction pathway in cardiomyocytes, we selected AC16-hCMs and WGA-coated MPs. By applying the MTS system to 2D cultured AC16-hCMs with and without WGA-coated MPs, we were able to observe the localization of the mechanoresponsive YAP protein in the nucleus and cytosol, respectively (Figure S4). To apply the MTS system to a physiological model, we evaluated cardiac hypertrophy markers. To this end, the MTS system was applied to AC16-hCMs with WGA-coated MPs under hypoxic conditions that have been demonstrated to induce cardiac hypertrophy (Figure 3). After incubation of AC16-hCMs under each condition, i.e., MPs only (control), MF only, hypoxia, and MPs with MF, for 24 h, we observed YAP protein localization by immunostaining and fluorescence microscopy (Figure 3A) and quantified the expression levels in the nucleus and cytosol (Figure 3B). The mechanoresponsive YAP protein was more strongly localized in the nucleus under MF conditions than under other conditions. Our analysis suggested that the MTS system is sufficient to stimulate a mechanotransduction pathway in AC16-hCMs, resulting in nuclear localization of the YAP protein.

We also performed RT-PCR analysis to measure the expression levels of cardiac hypertrophy markers when the MTS system stimulated a mechanotransduction pathway in AC16-hCMs (Figure 3C). Under our experimental conditions, the expression level of atrial natriuretic peptide (ANP), a physiological hypertrophy marker, was not enhanced by application of a MF only compared to that in the control group without an MF. However, the hypoxia groups both with and without MF-MPs exhibited increases of 1.5- and 1.3-fold, respectively, in the level of ANP. For β-myosin heavy chain (βMHC), a pathological hypertrophy marker, only the hypoxia group with MF-MPs showed a higher level of gene expression (4.7-fold) than the control group. Moreover, the level of cardiotrophin-1 (CT-1), a cardiac hypertrophy marker, was increased in the MF (1.3-fold), hypoxia (1.5-fold), and MF-MPs (2.2-fold) groups compared with the control group, while the expression level of this gene in the hypoxia group with MF-MPs was significantly higher than those in the MF and hypoxia groups (1.7- and 1.5-fold higher, respectively). Our results suggest that treatment with hypoxia and MF-MPs was sufficient to induce upregulation of these three cardiac hypertrophy markers.

**Figure 4:**
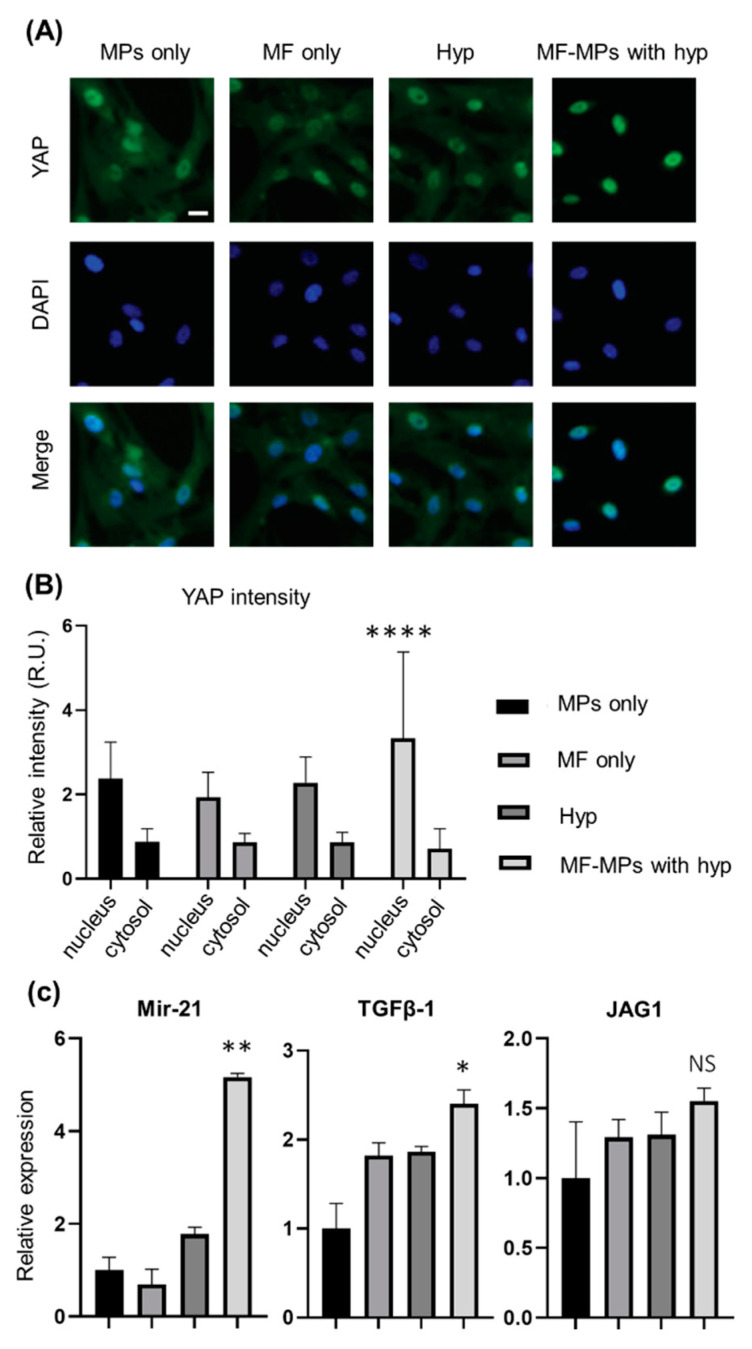
Effects of forces generated by the MTS system on protein localization and gene expression in hCF cells. Representative fluorescence images of YAP (green) and DAPI (blue) staining in hCF cells in the group of MPs only (control), MF only, hypoxia, and hypoxia with MF-MPs for 24 h (scale bars: 100 µm) (**A**). Quantitative analysis and comparison of YAP expression in the cytosol and nucleus of cultured hCF cells of each group (**B**). Quantitative mRNA expression data for cultured hCF cells in each group, as assessed by RT-PCR (**C**). Statistical analysis was conducted with a *t*-test to compare the control and MF-MPs with Hyp groups (****, *p* < 0.0001; **, *p* < 0.01; *, *p* < 0.05). There were two independent experiments, and RT-PCR analysis was duplicated.

Cardiac fibroblasts play a key role in cardiac hypertrophy via signaling interactions of growth factors and other molecules with cardiomyocytes. To evaluate whether conditions of cardiac hypertrophy influence the gene expression levels of representative markers in cardiac fibroblasts, we performed the same set of experiments described in Figure 4. Immunostaining and fluorescence microscopy showed that the nuclear YAP protein signal intensity was higher in the MF-MPs group than in the other groups, i.e., the control, MF only, and hypoxia groups. Quantification of the signals in the nucleus and cytosol showed that YAP proteins were more strongly localized in the nucleus, with increases of 1.4–1.7-fold in the MF-MPs group compared to the other groups. These results confirm that the MTS system was suitable for stimulating a mechanotransduction pathway in cardiac fibroblasts.

MicroRNA-21 (miR-21), transforming growth factor beta-1 (TGFβ-1), and Jagged1 (JAG1) were evaluated to determine whether conditions of cardiac hypertrophy and the MTS system can mediate gene expression in cardiac fibroblasts. First, we measured the expression level of miR-21, since an increased level of miR-21 is a marker of cardiac hypertrophy. Our analysis showed that the level of miR-21 in cardiac fibroblasts was 1.8- and 5.2-fold higher in the hypoxia group and MF-MPs group, respectively, than in the control group. This result suggests that the MTS system under hypoxic conditions significantly facilitates cardiac hypertrophy. The levels of the TGFβ-1 and JAG1 genes, which are affected by miR-21 and mechanical stress, were also evaluated in cardiac fibroblasts. The TGFβ-1 level was higher in the MF (1.8-fold), hypoxia (1.9-fold), and MF-MPs (2.4-fold) groups than in the control group, although the difference was statistically meaningful value only for the MF-MPs group (*p* ≤ 0.05). This result could be explained by close relationship of TGFβ-1 in cardiac fibroblasts to miR-21 and mechanical stress. For JAG1 gene expression, however, we found only a tendency toward a slightly increased level in the MF, hypoxia, and MF-MPs groups.

## 4. Discussion

Mechanotransduction is a key biological process for proper function of biological entities at any level [40]. In this study, by developing an MTS system through computational simulation, we showed that an MTS system can generate a uniform magnetic field across the whole area of a cell culture dish (35 mm). “Uniform” means that the magnetic flux density is almost constant at any position in the target region. Therefore, since our MTS system generates a small (or negligible) attractive magnetic force, the magnetic beads do not agglomerate even when the field rotates. Our findings suggest that application of a highly uniform force by many magnetic facilitates the establishment of a model of mechanically associated cardiac disease, i.e., cardiac hypertrophy. The experimental results verify the feasibility of the MTS system for mechanostimulation of cardiomyocytes and cardiac fibroblasts in vitro. However, our study had limitations in quantifying the force applied to each cell and the cell-to-cell variance. Thus, developing an electromagnetic stimulation system using Helmholtz coils, which can easily control the strength of the magnetic field and be expanded in 3D, might be valuable. 

The heart is a vital organ that supplies blood throughout the body by constant pumping action generated by synchronous contraction of cardiomyocytes [41]. This synchronous contraction is highly regulated by the interplay between cardiac cells such as cardiomyocytes and fibroblasts [42]. Therefore, cardiac cells are constantly exposed to forces in the cyclic contraction and relaxation modes, implying that mechanical loads in the myocardial microenvironment are central players in the physiological role of cardiomyocytes [43]. Thus, the development of the MTS system provides a unique approach to activating mechanotransduction pathways in biological systems, including cells and tissues. Simulation and estimation of the maximum torque generated by the MTS system could provide insight into exploring the mechanotransduction mechanisms of biological pathways. The advantage of the MTS system is its capability of transducing a highly uniform mechanical force into each cell and tissue. In addition, each MP can be engineered with specific ligands, allowing us to stimulate a target receptor alone or in combination with other receptors in mechanotransduction pathways.

We showed the effect of torque generated by MTS on heart cells by assessing two parameters: localization of a mechanosensitive protein and expression of hypertrophic and myofibroblastic markers. Nuclear localization of the YAP protein is a dramatic example of mechanotransduction in cells exposed to external forces. In our experiment, YAP proteins in cells subjected to torque under hypoxia were localized to the nucleus, indicating that this mechanotransduction pathway is activated by the MTS-CMP system. However, significantly less nuclear-localized YAP was seen in cells exposed to hypoxia or a magnetic field alone without generation of torque. Interestingly, hypoxic conditions activate nuclear localization of YAP, which leads to various effects, such as cell proliferation and growth [44,45]. In our experiment, we observed a significant increase in nuclear localization of YAP when torque was generated under hypoxia, while, for hypoxia without torque, nuclear localization of YAP was not observed in cardiomyocytes and fibroblasts. Further study is needed to elucidate the correlation between forces and hypoxia, because hypoxia is a key factor in various cardiac diseases, including myocardial infarction.

The gene expression of hypertrophic and myofibroblastic markers was evaluated in cardiomyocytes and fibroblasts subjected to torque. Hypertrophic markers such as ANP and β-MHC and myofibroblastic markers such as miR-21 and TGF-β were clearly induced by the torque generated by the MTS-CMP system in cardiomyocytes and fibroblasts, indicating that the force is transformed into biological signals. This result is consistent with that of a previous study showing that the expression of contractile and myofibroblast genes such as actin alpha 2 (ACTA2) was induced by static stretching of neonatal rat myocytes [28,46,47]. Interestingly, hypertrophic markers in CM and myofibroblastic markers in fibroblasts were synergistically overexpressed by application of the MTS system under hypoxic conditions. Although the gene expression of hypertrophic and myofibroblastic markers is induced under hypoxic conditions, the stimulation of cells by torque synergistically enhances the expression of these genes. Notably, the expression of CT-1, a marker of pathological cardiac hypertrophy, is increased under MTS in a hypoxic environment (Figure 3) but not under MTS alone (Figure S4). Although the expression of CT-1 is directly upregulated by hypoxia-inducible factor 1α (HIF-1α) in cardiomyocytes [48], the mechanism of the synergistic CT-1 expression by the combination of torque and hypoxia is controversial (Figure 3C). It is worthwhile to consider the fact that miR-21 and TGF-β are not only increased by mechanical stress, but also upregulated in cardiac hypertrophy [49,50]. Based on our findings, further study is needed to elucidate the molecular mechanisms underlying the interplay between cardiomyocytes and fibroblasts subjected to torque under hypoxia using the MTS system. One can hypothesize that mechanical stress induces overexpression of miR-21 and TGF-β in myofibroblasts, resulting in cardiac hypertrophy through cardiomyocyte-myofibroblast interactions. In addition, the evaluation of the protein level corresponding to genes would be further necessary to understand its physiological effects on cellular behavior, while a number of previous studies have shown the tight correlation of the protein level with gene level [51,52,53].

The versatility of the developed MTS system arises from the ability to modulate each component of the MTS, including the molecules conjugated to the MPs, which enables us to target specific cell receptors for activation, and the magnitude and frequency of the generated torque. In the current study, we utilized the WGA protein to target the cell surface because it binds to the glycosylated ectodomain of many membrane proteins, leading to the binding of MPs to the cell membrane. To target mechanosensitive molecules such as piezo1, MPs can be conjugated to an antibody specific for piezo1 in order to mediate the function of specific channel proteins. In addition, the torque can be modified by changing the magnets to electromagnets to modulate the magnetic flux density, modulating the rotation speed, and modifying the diameter of the MPs. Because of its versatility, for example, its ability to generate uniform and homogenous magnetic fields, transform these magnetic fields into torque using specifically targeted MPs, and be used for mesoscale experiments on cells or aggregates in 2D and 3D, the MTS system is an ideal tool for investigating mechanotransduction in biological research.

## 5. Conclusions

We developed an MTS system enabling us to accomplish highly parallel yet remote activation of mechanotransduction pathways in cardiomyocytes and cardiac fibroblasts. We showed that rotating MPs can supply a force sufficient to activate cardiac cells over long distances. Via the design of an efficient rotating magnetic field and engineered MPs, the capability of the MTS system could be extended to remote modulation of mechanotransduction pathways in cells. The MTS system could be further enhanced by the development of magnetic materials with higher magnetization values as well as advanced electromagnetic systems in order to achieve higher spatial and temporal resolutions. In addition, the use of biocompatible magnetic materials will benefit its clinical application. We believe that our approach, which not only is feasible across a long working distance but also does not require invasive implantation of an external stimulator, will facilitate the study of matters in basic mechanobiology and pathological disorders, including cardiac hypertrophy.

## Supplementary Materials

The following are available online at https://www.mdpi.com/2079-4991/10/9/1684/s1, Figure S1: Numerical simulation of MTS system by varying the distance between two magnets. magnetic flux density distribution from the simulation result and the streamline plots are shown., Figure S2: Number of MPs bound to each cell. Representative bright field image (A) of MPs bound to cells (scale bar: 100 µm). Histogram of the number of MPs (B) bound to single cells. Note that 5.8 ± 2.8 MPs were bound to single cells (*n* = 101)., Figure S3: Time capture images of MP strings in the MTS system rotating at 60 rpm. Images were acquired with Q-focus software (Euromex Microscope B.V., The Netherlands) using an Amscope microscope. The movement of the MP strings were aligned and rotated according to the rotational speed of the MTS system. Figure S4: Effects of forces generated by the MTS system on protein localization and gene expression in AC16 cells. Representative fluorescence images (A) of AC16 cells in the group with (MF-MPs) and without (MPs only) MTS application. Quantification of YAP expression in the cytosol and nucleus of cultured AC16 cells of each group (B). Quantitative mRNA expression data for genes related to cardiac hypertrophy in cultured AC16 cells of each group analyzed by RT-PCR (C). A *t*-test was used to compare the MPs only with MF-MPs groups (*, *p* < 0.05). Video S1: Video of the response of MP strings in the MTS system rotating at 60 rpm. MP strings were aligned and rotated according to the rotational speed of the MTS system.
