# Supplementary material for: Development of Magnetic Torque Stimulation (MTS) Utilizing Rotating Uniform Magnetic Field for Mechanical Activation of Cardiac Cells
Source: Nanomaterials (Basel). 2020 Aug 27;10(9):1684. doi: 10.3390/nano10091684 (PMC7557977; doi:10.3390/nano10091684)
Supplement: Supplementary file 1 [file nanomaterials-10-01684-s001.zip › SI file/SI_final.pdf]

Supporting information for

Development of magnetic torque stimulation (MTS) utilizing rotating uniform magnetic field for mechanical activation of cardiac cells

Myeongjin Song[§,†], *Jongseong Kim*[§,†], *Hyundo Shin*[‡], *Yekwang Kim*[†], *Hwanseok Jang*[†],

*Yongdoo Park**[,†], *Seung-Jong Kim**[,†]

[†]Department of Biomedical Sciences, College of Medicine, Korea University, Seoul 02841, Republic of Korea

[‡]Department of Mechanical Engineering, Yonsei University, Seoul 03722, Republic of Korea

[§]These authors contributed equally

E-mail: ydpark67@korea.ac.kr and sjkim586@korea.ac.kr

Figure S1, S2, S3, S4

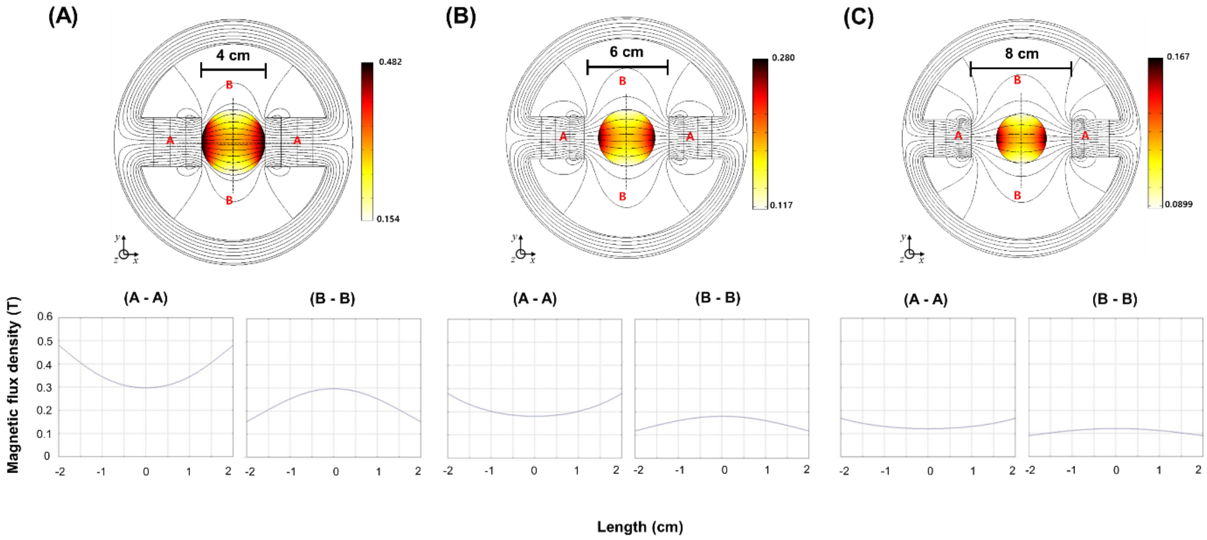

Figure S1. Numerical simulation of MTS system by varying the distance between two magnets. magnetic flux density distribution from the simulation result and the stream line plots are shown.

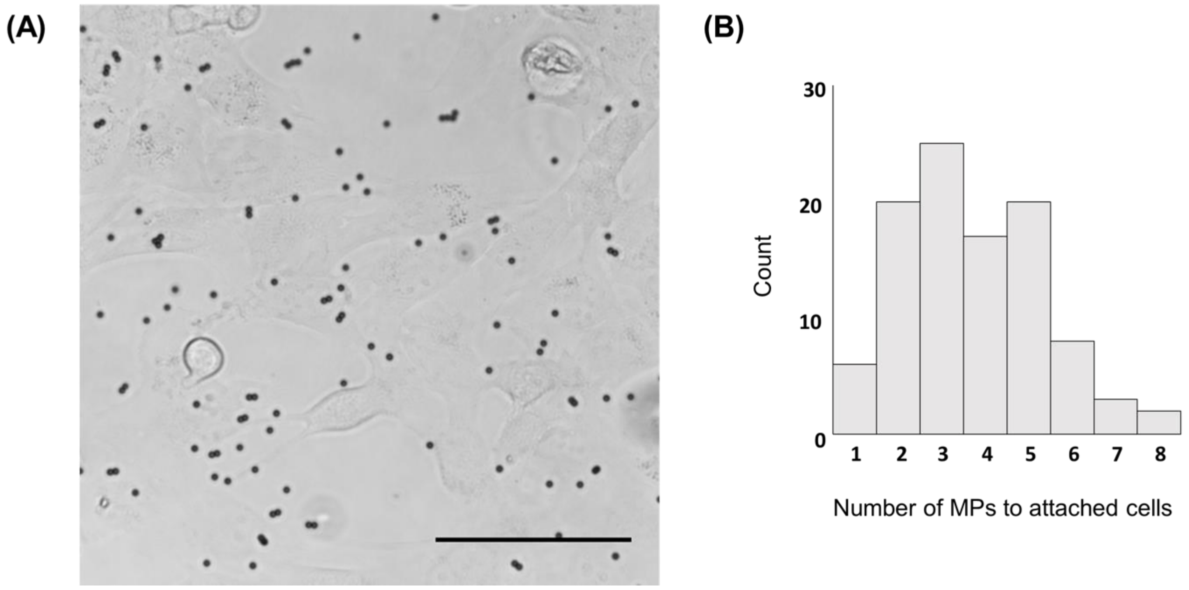

Figure S2. Number of MPs bound to each cell. Representative bright field image (A) of MPs bound to cells (scale bar: 100 μm). Histogram of the number of MPs (B) bound to single cells. Note that 5.8±2.8 MPs were bound to single cells (n = 101).

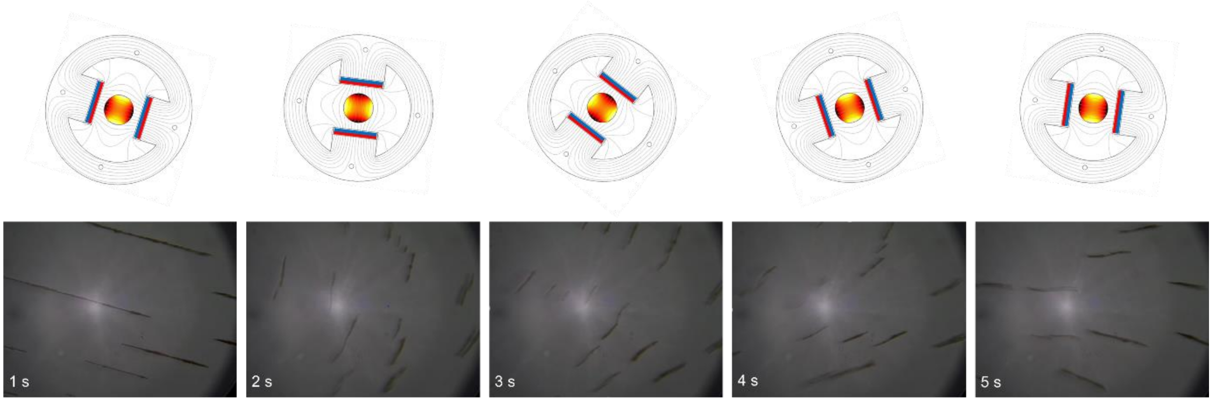

Figure S3. Time capture images of MP strings in the MTS system rotating at 60 rpm. Images were acquired with Q-focus software (Euromex Microscope B.V., The Netherlands) using an Amscope microscope. The movement of the MP strings were aligned and rotated according to the rotational speed of the MTS system.

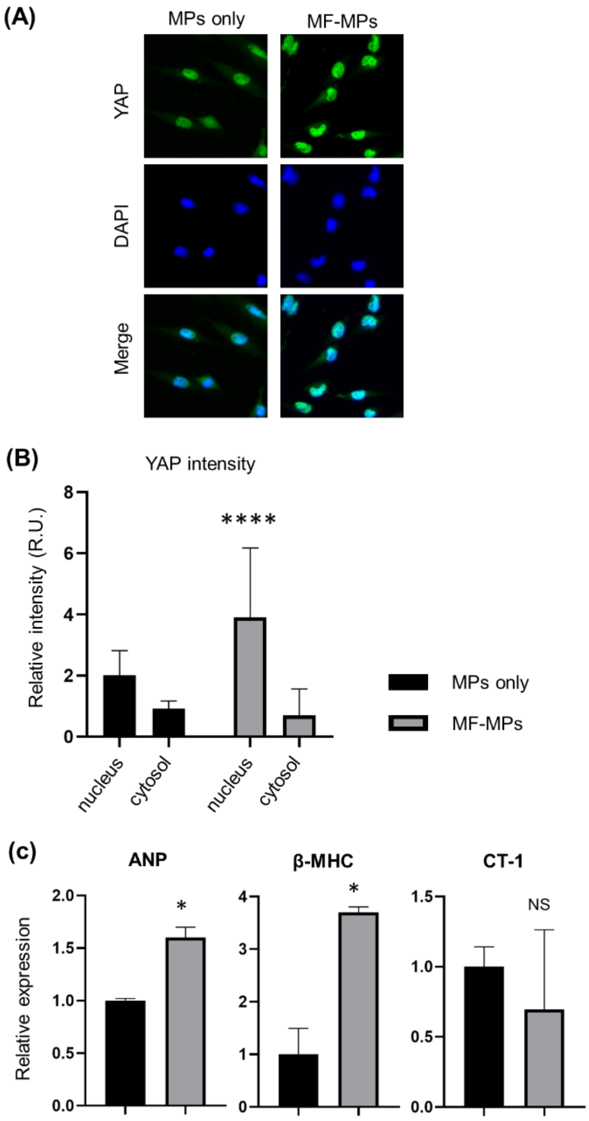

Figure S4. Effects of forces generated by the MTS system on protein localization and gene expression in AC16 cells. Representative fluorescence images (A) of AC16 cells in the group with (MF-MPs) and without (MPs only) MTS application. Quantification of YAP expression in the cytosol and nucleus of cultured AC16 cells of each group (B). Quantitative mRNA expression data for genes related to cardiac hypertrophy in cultured AC16 cells of each group analyzed by RT-PCR (C). A t-test was used to compare the MPs only with MF-MPs groups (*, $P<0.05$).

Supplement movie 1. Video of the response of MP strings in the MTS system rotating at 60 rpm. MP strings were aligned and rotated according to the rotational speed of the MTS system.
